# The relationship among emotion regulation, emotional labor, and job burnout in clinical nurses

**DOI:** 10.3389/fpubh.2026.1928871

**Published:** 2026-09-23

**Authors:** Mi Zhou, Hongzhi Wang, Huan He, Hongliang Dai, Zanru Yang

**Affiliations:** 1 School of Nursing, Jinzhou Medical University, Jinzhou, Liaoning, China; 2 The Third Affiliated Hospital of Jinzhou Medical University, Jinzhou, Liaoning, China; 3 The First Affiliated Hospital of Jinzhou Medical University, Jinzhou, Liaoning, China

**Keywords:** clinical nurses, cross-sectional study, emotional labor, emotional regulation, job burnout

## Abstract

**Objective:**

To explore the cross-sectional associations among emotional regulation strategies, emotional labor, and job burnout among clinical nurses in a single-center setting, and to examine whether cognitive reappraisal moderates the association between surface acting and job burnout, thereby providing a basis for subsequent multi-center longitudinal studies and nursing management research.

**Methods:**

A single-center, convenience-sampled cross-sectional correlational study was conducted, enrolling 210 clinical nurses from a tertiary hospital in Jiangsu Province, China. Measures included a demographic questionnaire, the Emotion Regulation Questionnaire (ERQ), the Emotional Labor Scale (ELS), and the Maslach Burnout Inventory–Health Services Survey (MBI-HSS). Analyses were conducted using descriptive statistics, Pearson correlation analysis, one-way analysis of variance (ANOVA) with Tukey’s HSD *post hoc* comparisons, stratified multiple linear regression, and interaction term regression. Regression results included unstandardized coefficients (B), standardized coefficients (β), 95% confidence intervals, and diagnostics for multicollinearity.

**Results:**

Cognitive reappraisal was negatively correlated with burnout (*r* = −0.628, *p* < 0.001), while expressive suppression was positively correlated with it (*r* = 0.561, *p* < 0.001). Surface acting was strongly positively correlated (*r* = 0.714, *p* < 0.001), while deep acting (*r* = −0.481, *p* < 0.001) and emotional expression requirements (*r* = −0.344, *p* < 0.001) were negatively correlated with burnout. Regression analysis revealed that surface acting, cognitive reappraisal, expressive suppression, deep acting, and emotional expression requirements were all independently associated with the composite burnout score. Moderation analysis revealed that the interaction term between surface acting and cognitive reappraisal was statistically significant (B = −0.075, 95% CI: −0.115 to −0.035, *p* < 0.001), with a unique incremental explanation rate of Δ*R*^2^ = 0.015.

**Conclusion:**

Among clinical nurses, cognitive reappraisal, deep acting, and emotional expression requirements were associated with lower composite scores for job burnout, while expressive suppression and surface acting were associated with higher burnout scores.

## 1 Introduction

Nursing burnout is no longer viewed merely as an individual psychological distress but is increasingly recognized as a core issue concerning patient safety, nursing quality, and the resilience of the healthcare system. Nursing burnout is robustly associated with an increase in patient safety incidents, decreased patient satisfaction, and a deterioration in subjective evaluations of nursing quality (1). In professional settings with high emotional demands, nursing staff must constantly navigate the tension between institutional requirements, professional standards, and their genuine personal feelings. Particularly in contexts such as pain management, critical care, end-of-life care, and conflict resolution, emotional labor is virtually an integral part of clinical practice itself. Recent reviews on the scope of nurses’ emotional labor indicate that it not only affects individuals’ feelings of fatigue, job satisfaction, and intention to leave but is also closely related to team atmosphere, doctor-patient relationships, and organizational performance (2).

From a theoretical perspective, the Job Demands-Resources (JD-R) theory provides a robust analytical framework for understanding nurses’ emotional labor and burnout. This theory emphasizes that when job demands consistently exceed the resources available to an individual, employees are more likely to enter a state of exhaustion; conversely, psychological resources such as organizational support, self-efficacy, and emotional regulation skills can mitigate this process (3). The job demands of nursing positions include not only shifts, workload, complexity of patient conditions, and exposure to risk, but also implicit demands requiring constant control over facial expressions, tone of voice, attitude, and emotional cues. In other words, the occupational stress faced by nurses is not merely about the volume of tasks performed, but also about the emotional manner in which those tasks are carried out.

Emotional regulation refers to an individual’s ability to identify, evaluate, and modify emotional experiences and their outward expressions, with cognitive reappraisal and expressive suppression being the two most common process dimensions (4). Cognitive reappraisal typically occurs relatively early in the emotional response and emphasizes reinterpreting the meaning of an event; expressive suppression often occurs later in the emotional response and focuses on suppressing already generated facial expressions and external reactions. Extensive psychological evidence indicates that positive cognitive reappraisal is associated with greater psychological resilience, lower perceived stress, and higher subjective well-being, whereas overreliance on suppression leads to increased cognitive load and emotional exhaustion (5, 6). For nurses, this distinction has more direct professional implications: when nurses are able to reinterpret patient behavior, family conflicts, and work-related events, emotional labor is transformed into an integral part of professional interactions; conversely, when nurses frequently suppress genuine emotions without engaging in the process of meaning-making, emotional labor is more likely to evolve into emotional dysregulation and job burnout.

Emotional labor refers to the process by which individuals regulate their internal emotional experiences and external expressions to meet the demands of an organizational or professional role. This study employed the Chinese version of the Emotional Labor Scale (ELS) developed by Luo Hong et al. (2008)^,^ which was adapted from Grandey’s (2003) Emotional Labor Scale to reflect the Chinese nursing context (7, 8). The scale measures three types of emotional labors: surface acting, deep acting, and emotional expression requirements. Among these, surface acting reflects the extent to which an individual meets job requirements by adjusting their external expressions and behaviors. Deep acting reflects the extent to which individuals proactively adjust their internal feelings to align with the occupational context. Emotional expression requirements primarily reflect the degree to which hospitals, departments, or nursing professional roles regulate nurses’ service attitudes, communication styles, and emotional displays (7).

Although there is already a substantial body of evidence linking nurses’ emotional labor to job burnout, several issues warrant further investigation. First, while some studies treat emotional regulation as a background variable, few simultaneously examine the differential associations between cognitive reappraisal, expressive suppression, and various dimensions of emotional labor. Second, in previous studies, surface acting has typically been associated with higher levels of burnout, whereas the direction and strength of the association between deep acting and emotional expression requirements are influenced by factors such as department characteristics, age structure, organizational culture, and supportive environments (8–13). Third, existing research has shown that cognitive reappraisal alters the strength of the association between surface acting and job burnout (14). However, its stability and temporal sequence still require further validation through longitudinal or experimental studies.

Based on this, the present study takes emotional regulation in the nursing profession as its entry point and employs a cross-sectional correlational design to analyze the associations among clinical nurses’ emotional regulation strategies, emotional labor, and job burnout, while exploring whether cognitive reappraisal moderates the statistical association between surface acting and job burnout.

## 2 Participants and methods

### 2.1 Study participants

This study employed a single-center, cross-sectional correlational design and recruited clinical nurses from a tertiary hospital in Jiangsu Province, China via convenience sampling. The study aimed to describe the statistical associations among the variables at a single point in time and did not involve any training or nursing interventions. The survey adhered to the principles of voluntary participation, anonymous completion, and confidentiality of information. Each participant provided written informed consent. This study complies with the Declaration of Helsinki and was reviewed and approved by the Ethics Committee of Jinzhou Medical University (Ethics Approval No. JZMULL2026100).

### 2.2 Inclusion and exclusion criteria

#### 2.2.1 Inclusion criteria

Participants in this study should meet all of the following criteria:

① Holding a valid nursing license and be engaged in frontline nursing work in clinical departments at the study hospital;
② Having worked continuously in their current position for ≥6 months, enabling a relatively stable reflection of emotional labor and job burnout in clinical nursing roles;
③ Possessing the ability to independently read and understand the questionnaire content and complete the questionnaire on their own;
④ Aware of the study’s purpose, content, and data confidentiality principles, and voluntarily participate in the study.

#### 2.2.2 Exclusion criteria

Participants meeting any of the following criteria will be excluded:

① Nurses in non-clinical frontline positions, such as those in the Nursing Department, administrative management, logistics, or the supply room;
② Nurses who, during the survey period, are on advanced training, residency rotations, long-term sick leave, maternity leave, personal leave, or are otherwise away from clinical duties;
③ Nursing interns, nursing assistants, and individuals who have not obtained a nursing license;
④ Individuals who have recently experienced major life events, severe physical illnesses, or mental health conditions that may affect the accuracy of their responses.

#### 2.2.3 Sample size calculation

This study primarily employs correlation analysis and multiple linear regression. During the study design phase, based on common empirical rules for main-effects regression—estimating approximately 13 primary predictor variables with 10 to 15 cases per variable—the required sample size was estimated to be approximately 130 to 195 cases. Taking into account invalid questionnaires, missing data, and model stability, the planned sample size was set at 200 or more cases. This empirical estimate did not specifically conduct a prior power analysis for interaction effects. Therefore, moderation effects are considered exploratory analyses. A *post hoc* sensitivity analysis was conducted based on the final sample size of n = 210. Under conditions of α = 0.05, one incremental interaction term, and a full covariate model, 80% statistical power is sufficient to detect an incremental f^2^ ≈ 0.038. The observed incremental f^2^ for the interaction term in this study was 0.070. This sensitivity analysis was used solely to illustrate the sample’s ability to detect the observed magnitude of the interaction effect.

### 2.3 Measures

#### 2.3.1 Demographic questionnaire

The Demographic Questionnaire includes gender, age, marital status, educational background, department, years of service, professional title, employment status, position, weekly working hours, and monthly frequency of night shifts.

#### 2.3.2 Emotion regulation questionnaire

Emotional regulation ability was measured using the Emotion Regulation Questionnaire (ERQ)[4]. The scale consists of 10 items, including 6 items for cognitive reappraisal and 4 items for expressive suppression, using a 7-point rating scale. Higher scores indicate a stronger tendency to use the corresponding emotional regulation strategy. The original study by Gross and John confirmed that the ERQ has a clear two-dimensional structure (4). The structural validity and usability of the Chinese version of the ERQ was also validated in a Chinese-speaking sample (15). In the present study, the Cronbach’s α coefficients for the Cognitive Reappraisal and Expressive Suppression subscales were 0.896 and 0.829, respectively, indicating good internal consistency.

#### 2.3.3 Emotional labor scale

Emotional labor of nurses was measured using the Chinese version of the ELS (C-ELS)[8]in this study, which was adapted from the original version developed by Grandey (7). The C-ELS has three dimensions of surface acting (7 items), deep acting (3 items), and emotional expression requirements (4 items). Surface acting reflects the extent to which an individual meets job requirements by adjusting their external expressions and behaviors. Deep acting reflects the extent to which individuals proactively adjust their internal feelings to align with the occupational context. As for emotional expression requirements reflect the extent to which the hospital or position regulates nurses’ emotional displays, service attitudes, and communication behaviors. Each item is rated using a 6-point Likert scale, with higher scores indicating more higher level of emotional labor. The Cronbach’s α values for surface acting, deep acting, and emotional expression requirements were 0.884, 0.820, and 0.827, respectively.

#### 2.3.4 Burnout scale

Job burnout was measured using the 22-item Maslach Burnout Inventory-Human Services Survey (MBI-HSS) (16), which includes 9 items for emotional exhaustion, 5 items for depersonalization, and 8 items for personal accomplishment. Each item is scored on a Likert scale from 0 to 6. Higher scores on emotional exhaustion and depersonalization indicate more pronounced burnout symptoms, while lower scores on personal accomplishment indicate a higher degree of low personal accomplishment. Given the relative conceptual independence of the three dimensions of the MBI-HSS, this study not only reports the three subscales separately but also, to adhere to the predefined statistical plan, converts the Personal Accomplishment score (based on the 48-point raw scale) to “Low Personal Accomplishment” and adds it to the Emotional Exhaustion and Depersonalization scores to form the “Research-Based Burnout Composite Score.” This composite score serves solely as an exploratory overall indicator and is not considered the standard diagnostic total score of the MBI-HSS. To test the robustness of the conclusions regarding the composite indicator, sensitivity analyses were conducted using the three subscales as separate outcomes. In the study sample, the Cronbach’s α coefficients for emotional exhaustion, depersonalization, and personal accomplishment were 0.911, 0.808, and 0.851, respectively. The composite internal consistency α for the 22 items with unified direction was 0.948.

### 2.4 Data collection method

This study employed convenience sampling to conduct an anonymous questionnaire survey across various clinical departments at a tertiary hospital in Jiangsu Province. Researchers uniformly explained the study objectives, informed consent, voluntary participation, and confidentiality principles. Participants independently completed the demographic information form, ERQ, ELS, and MBI-HSS, which took approximately 10–15 min to complete. After the questionnaires were collected, they were first checked for completeness and logical consistency. Questionnaires with missing items, repetitive or pattern-based responses, duplicate entries, or obvious logical contradictions were excluded. The 210 valid questionnaires ultimately included in the analysis had no remaining missing values at the scale item level.

### 2.5 Quality control

This study implemented quality control measures during three phases: pre-survey, during the survey, and post-survey. Prior to the survey, the questionnaire instructions and survey procedures were standardized according to the research protocol, and a pilot survey was conducted to assess item comprehensibility. During the survey, researchers who had undergone standardized training administered the questionnaires, emphasizing anonymity, voluntariness, and independent responses to minimize social desirability bias; after the survey, two researchers cross-checked the data, reviewed outliers, missing values, and logical inconsistencies, and strictly calculated dimension scores and total scores in accordance with the scoring rules for each scale.

### 2.6 Statistical methods

Statistical analysis was performed using SPSS 27.0 software. Continuous variables are expressed as mean ± standard deviation, and categorical variables are presented as frequencies and percentages. One-way analysis of variance (ANOVA) was used to compare different age groups, departments, professional titles, and night-shift groups. When differences were statistically significant, Tukey’s HSD *post hoc* tests were conducted. An independent-sample t-test was used to compare employment types. Pearson correlation analysis was used to examine the relationships among emotional regulation, emotional labor, and job burnout. Hierarchical multiple linear regression was performed with the composite job burnout score as the dependent variable, sequentially including demographic and job characteristics, emotional regulation variables, and emotional labor variables. Unstandardized coefficients (B), standardized coefficients (β), 95% confidence intervals (95% CI), and variance inflation factor (VIF) were reported. Prior to moderation analysis, surface acting and cognitive reappraisal were mean-centered. Interaction terms were constructed, and simple slope analysis was performed. The regression models were tested for residual normality, homoscedasticity, linearity, independence of errors, and multicollinearity. Harman’s single-factor test was used to preliminarily assess common method bias. A two-sided *p* < 0.05 was considered statistically significant.

## 3 Results

### 3.1 General characteristics

A total of 230 questionnaires were distributed, with 220 returned, yielding a response rate of 95.65%. After verification, 10 invalid questionnaires were excluded due to serious omissions, duplicate responses, or obvious logical contradictions. Ultimately, 210 valid questionnaires were included, representing a validity rate of 95.45% and a valid response rate of 91.30%. The overall quality of the questionnaires was good and met the requirements for subsequent statistical analysis.

This study included a total of 210 clinical nurses, of whom 204 were female (97.1%);100 (47.6%) were aged 30–39; 154 (73.3%) were married; and 170 (81.0%) held a bachelor’s degree. Many of them worked in internal medicine (27.1%) and surgery (21.0%), while 39 nurses (18.6%) worked in intensive care units or emergency departments. The mean age of the sample was (32.82 ± 6.76) years, the mean length of service was (10.42 ± 6.77) years, the mean weekly working hours were (44.78 ± 4.62) hours, and the mean number of night shifts per month was (3.97 ± 2.27). Detailed information is shown in Table 1.

**Table 1:** Analysis of general characteristics (*n* = 210).

Variable	Category	*n*	Percentage (%)
Gender	Male	6	2.9
	Female	204	97.1
Age group (years)	≤29	75	35.7
	30–39	100	47.6
	≥40	35	16.7
Marital status	Unmarried	51	24.3
	Married	154	73.3
	Other	5	2.4
Highest level of education	Associate’s degree	40	19.0
	Bachelor’s degree	170	81.0
Department	Internal medicine	57	27.1
	Surgical system	44	21.0
	ICU/emergency department	39	18.6
	Obstetrics/pediatrics	33	15.7
	Outpatient/other	37	17.6
Professional title	Registered nurse	22	10.5
	Junior nurse	78	37.1
	Nurse-in-charge	92	43.8
	Associate chief nurse and above	18	8.6
Employment type	Contract-based	70	33.3
	Permanent staff/long-term contracts	140	66.7
Positions	Clinical Nurse	180	85.7
	Team leader/clinical instructor	30	14.3
Monthly night shift (times)	0–2	61	29.0
	3–5	96	45.7
	≥6	53	25.2

### 3.2 Common method bias test

All 46 self-report items from the ERQ, ELS, and MBI-HSS were simultaneously included in Harman’s single-factor test. KMO = 0.940. Bartlett’s sphericity test *χ*^2^ = 5,464.398, df = 1,035, *p* < 0.001. Unrotated principal component analysis identified 6 factors with eigenvalues > 1, explaining a cumulative 59.016% of the variance, with the first factor explaining 36.287%, which is below the empirical threshold of 40% (Tables 2, 3). These results did not show clear signs of a single dominant factor. However, it is worth noting that the Harman test is only of rough diagnostic significance and cannot be used to rule out common method variance, especially when all variables are derived from self-report questionnaires administered at the same time point.

**Table 2:** KMO coefficient and Bartlett’s sphericity test results.

Item	Result
KMO Adequacy Statistic	0.940
Approximate chi-square value for Bartlett’s sphericity test	5464.398
Degrees of freedom for Bartlett’s sphericity test	1,035
Significance of Bartlett’s sphericity test	<0.001

**Table 3:** Total variance explained in Harman’s single-factor test.

Factor	Initial eigenvalue	Percentage of variance explained (%)	Cumulative percentage of variance explained (%)
1	16.692	36.287	36.287
2	3.253	7.072	43.359
3	2.221	4.827	48.186
4	1.888	4.104	52.290
5	1.697	3.688	55.978
6	1.397	3.038	59.016

The principal component analysis method used did not involve rotation.

### 3.3 Current status of emotional regulation ability, emotional labor, and burnout

As shown in Table 4, the 210 clinical nurses scored (23.98 ± 4.36) on cognitive reappraisal and (16.05 ± 2.90) on expressive suppression. Regarding emotional labor, the scores for surface acting, deep acting, and emotional expression requirements were (24.47 ± 4.82), (10.47 ± 2.28), and (13.99 ± 2.93), respectively, with a total emotional labor score of (48.92 ± 5.38). The results indicate that the study participants faced varying degrees of demands for internal emotional regulation, external emotional management, and organizational emotional display in their nursing work. Among the three dimensions of the MBI-HSS, the scores for emotional exhaustion, depersonalization, and personal accomplishment were (27.01 ± 6.18), (15.09 ± 3.37), and (23.94 ± 5.04), respectively. The composite score for research-based occupational burnout, constructed according to the predetermined methodology, was (66.16 ± 13.69).

**Table 4:** Scores for emotional regulation ability, emotional labor, and job burnout among clinical nurses.

Variable	Mean	Standard Deviation	Minimum	Maximum
Cognitive reappraisal	23.98	4.36	13	35
Expressive suppression	16.05	2.90	9	22
Surface acting	24.47	4.82	12	36
Deep acting	10.47	2.28	4	17
Emotional expression requirements	13.99	2.93	8	23
Total emotional labor score	48.92	5.38	34	63
Emotional exhaustion	27.01	6.18	14	46
Depersonalization	15.09	3.37	8	25
Sense of personal accomplishment	23.94	5.04	5	37
Overall burnout score	66.16	13.69	34	114

As shown in Table 5, one-way ANOVA revealed significant differences by age group (*F* = 10.742, *p* < 0.001), department (*F* = 9.426, *p* < 0.001), professional title (*F* = 3.818, *p* = 0.011), employment status (*t* = 2.490, *p* = 0.014), and monthly night shift (*F* = 26.316, *p* < 0.001) in job burnout. Since there were only 6 male participants, this study did not conduct inferential tests between groups for gender. The gender distribution is presented for descriptive purposes only. Tukey HSD *post hoc* comparisons showed that, regarding age, the group aged ≥40 had lower scores than the 30–39 age group (*P_adj_* = 0.006) and the ≤29 age group (*P_adj_* < 0.001) in job burnout. As for department, the ICU/Emergency group had higher scores than Internal Medicine (*P_adj_* = 0.002), Surgery (*P_adj_* < 0.001), Obstetrics/Pediatrics (*P_adj_* < 0.001), and Outpatient/Other (*P_adj_* < 0.001) in job burnout. For professional title, associate chief nurses and above group had lower scores than junior nurses (*P_adj_* = 0.025) and nurses-in-charge (*P_adj_* = 0.032). Regarding night shifts, the group working ≥6 times per month had higher scores than the 0–2 times per month and 3–5 times per month groups (both *P_adj_*s < 0.001). The remaining pairwise comparisons did not reach statistical significance.

**Table 5:** Comparison of burnout scores across groups.

Variable	Group	*n*	Composite job burnout score	Statistical value	*p*
Age Group (years)	≤29	75	70.29 ± 13.91	*F* = 10.742	<0.001
	30–39	100	65.95 ± 13.32		
	≥40	35	57.89 ± 10.27		
Department	Internal medicine	57	66.67 ± 12.59	*F* = 9.426	<0.001
	Surgical system	44	62.95 ± 11.98		
	ICU/emergency department	39	76.51 ± 14.71		
	Obstetrics/pediatrics	33	63.85 ± 13.54		
	Outpatient/other	37	60.32 ± 10.45		
Professional title	Registered nurse	22	70.82 ± 14.10	*F* = 3.818	0.011
	Junior nurse	78	68.33 ± 13.76		
	Nurse-in-charge	92	64.66 ± 13.61		
	Associate chief nurse and above	18	58.67 ± 9.37		
Employment type	Contract-based	70	69.41 ± 13.28	*t* = 2.490	0.014
	Permanent/long-term contract	140	64.53 ± 13.64		
Monthly night shift (times)	0–2	61	60.89 ± 11.54	*F* = 26.316	<0.001
	3–5	96	63.78 ± 11.18		
	≥6	53	76.53 ± 14.78		

### 3.4 Analysis of the correlation between clinical nurses’ emotional regulation ability, emotional labor, and job burnout

Pearson correlation analysis showed that cognitive reappraisal was negatively correlated with the composite score of research-based job burnout (*r* = −0.628, 95% CI: −0.703 to −0.538, *p* < 0.001), while expressive suppression was positively correlated with the composite score for job burnout (*r* = 0.561, 95% CI: 0.461 to 0.648, *p* < 0.001).

Among the dimensions of emotional labor, surface acting was strongly positively correlated with the composite score for job burnout (*r* = 0.714, 95% CI: 0.641–0.775, *p* < 0.001). Deep acting was negatively correlated with the composite score for job burnout (*r* = −0.481, 95% CI: −0.579 to −0.370, *p* < 0.001). Emotional expression requirements were also negatively correlated with the composite score for job burnout (*r* = −0.344, 95% CI: −0.458 to −0.219, *p* < 0.001). This indicates that the direction of the association between different dimensions of emotional labor and occupational burnout varies.

Cognitive reappraisal was negatively correlated with surface acting (*r* = −0.391, *p* < 0.001) and positively correlated with deep acting (*r* = 0.388, *p* < 0.001) and emotional expression requirements (*r* = 0.310, *p* < 0.001). Expressive suppression was positively correlated with surface acting (*r* = 0.470, *p* < 0.001) and negatively correlated with deep acting (*r* = −0.212, *p* = 0.002) and emotional expression requirements (*r* = −0.207, *p* = 0.003). The correlation coefficients and 95% CIs are shown in Table 6.

**Table 6:** Pearson correlation analysis of clinical nurses’ emotional regulation, emotional labor, and total burnout scores.

Variable 1	Variable 2	*r*	95% CI	*p*
Cognitive reappraisal	Expressive suppression	−0.342	[−0.456, −0.217]	<0.001
Cognitive reappraisal	Surface acting	−0.391	[−0.500, −0.270]	<0.001
Cognitive reappraisal	Deep acting	0.388	[0.266, 0.497]	<0.001
Cognitive reappraisal	Emotional expression requirements	0.310	[0.182, 0.428]	<0.001
Cognitive reappraisal	Total burnout score	−0.628	[−0.703, −0.538]	<0.001
Expressive suppression	Surface acting	0.470	[0.358, 0.569]	<0.001
Expressive suppression	Deep acting	−0.212	[−0.337, −0.078]	0.002
Expressive suppression	Emotional expression requirements	−0.207	[−0.333, −0.074]	0.003
Expressive suppression	Total burnout score	0.561	[0.461, 0.648]	<0.001
Surface acting	Deep acting	−0.309	[−0.427, −0.181]	<0.001
Surface acting	Emotional expression requirements	−0.155	[−0.284, −0.020]	0.025
Surface acting	Total burnout score	0.714	[0.641, 0.775]	<0.001
Deep acting	Emotional expression requirements	0.231	[0.098, 0.355]	<0.001
Deep acting	Total burnout score	−0.481	[−0.579, −0.370]	<0.001
Emotional expression requirements	Total burnout score	−0.344	[−0.458, −0.219]	<0.001

95% CI calculated using Fisher’s z-transformation. All p-values are from two-tailed tests.

### 3.5 Analysis of factors influencing job burnout

A Hierarchical multiple linear regression model was constructed with the composite score for research-related job burnout as the dependent variable. To avoid over-compressing departmental information, departments were included in the model as k-1 dummy variables, with the Internal Medicine Department serving as the reference group. Model 1 included age, the number of night shifts per month, gender, employment type, and 4 departmental dummy variables. *R*^2^ = 0.395, adjusted *R*^2^ = 0.371 (*F* = 16.437, *p* < 0.001). Age was negatively associated with the composite burnout score (*B* = −0.698, *β* = −0.344, *p* < 0.001), while the number of monthly night shifts was positively associated (*B* = 2.930, *β* = 0.485, *p* < 0.001). The remaining covariates were not statistically significant (Table 7).

**Table 7:** Hierarchical multiple linear regression analysis of the composite burnout score among clinical nurses.

Model	Variables	*B*	SE	95% CI	*β*	*t*	*p*	VIF
Model 1	Constant	70.785	6.302	[58.359, 83.211]		11.232	<0.001	
	Age	−0.698	0.112	[−0.919, −0.476]	−0.344	−6.204	<0.001	1.03
	Monthly night shift	2.930	0.461	[2.021, 3.839]	0.485	6.358	<0.001	1.94
	Gender (female = 1)	5.283	4.582	[−3.752, 14.319]	0.064	1.153	0.250	1.04
	Employment type (contract-based = 1)	3.134	1.631	[−0.082, 6.351]	0.108	1.922	0.056	1.05
	Department (surgical *vs*. medical)	−1.762	2.206	[−6.111, 2.587]	−0.053	−0.799	0.425	1.44
	Department (ICU/emergency *vs*. internal medicine)	2.532	2.532	[−2.460, 7.525]	0.072	1.000	0.318	1.73
	Department (obstetrics/pediatrics *vs*. internal medicine)	−0.867	2.401	[−5.600, 3.867]	−0.023	−0.361	0.718	1.36
	Department (outpatient/other *vs*. internal medicine)	2.881	2.621	[−2.287, 8.049]	0.080	1.099	0.273	1.78

As shown in Table 8, Model 2 further included cognitive reappraisal and expressive suppression, increasing *R*^2^ to 0.656 (adjusted *R*^2^ = 0.639), representing an increase of Δ*R*^2^ = 0.261 compared to Model 1, corresponding to an incremental Cohen’s *f*^2^ of 0.759. Cognitive reappraisal was negatively associated with the composite score of job burnout (*B* = −1.415, *β* = −0.450, 95% CI: −1.713 to −1.116, *p* < 0.001), while expressive suppression was positively associated (*B* = 1.249, *β* = 0.265, 95% CI: 0.795 to 1.702, *p* < 0.001). The coefficients for age and number of night shifts decreased compared to Model 1. However, this reduction in coefficients represents only a descriptive change following stratification and cannot be taken as evidence of a mediating effect.

**Table 8:** Hierarchical multiple linear regression analysis of the composite score for clinical nurse burnout.

Model	Variables	*B*	SE	95% CI	*β*	*t*	*p*	VIF
Model 2	Constant	74.425	6.978	[60.665, 88.185]		10.666	<0.001	
	Age	−0.208	0.094	[−0.394, −0.022]	−0.103	−2.208	0.028	1.26
	Monthly night shift	1.855	0.372	[1.121, 2.589]	0.307	4.983	<0.001	2.20
	Gender (female = 1)	3.208	3.477	[−3.649, 10.064]	0.039	0.923	0.357	1.04
	Employment type (contract-based = 1)	2.209	1.248	[−0.253, 4.671]	0.076	1.770	0.078	1.08
	Department (surgical *vs*. medical)	−1.028	1.673	[−4.327, 2.271]	−0.031	−0.614	0.540	1.44
	Department (ICU/emergency *vs*. internal medicine)	3.645	1.931	[−0.162, 7.452]	0.104	1.888	0.060	1.75
	Department (obstetrics/pediatrics *vs*. internal medicine)	2.575	1.846	[−1.065, 6.215]	0.069	1.395	0.165	1.40
	Department (outpatient/other *vs*. internal medicine)	2.120	1.991	[−1.806, 6.046]	0.059	1.065	0.288	1.79
	Cognitive reappraisal	−1.415	0.151	[−1.713, −1.116]	−0.450	−9.354	<0.001	1.34
	Expressive suppression	1.249	0.230	[0.795, 1.702]	0.265	5.432	<0.001	1.37

After adding surface acting, deep acting, and emotional expression requirements to Model 3, as shown in Table 9, *R*^2^ = 0.769 and adjusted *R*^2^ = 0.754, representing an increase of Δ*R*^2^ = 0.113 (incremental *f*^2^ = 0.487) compared to Model 2. The results showed that surface acting was positively associated with total job burnout scores (*B* = 0.958, *β* = 0.337, 95% CI: 0.698–1.218, *p* < 0.001), while cognitive reappraisal (*B* = −0.941, *β* = −0.299, 95% CI: −1.207 to −0.675, *p* < 0.001), deep acting (*B* = −1.018, *β* = −0.169, 95% CI: −1.479 to −0.558, *p* < 0.001), emotional expression requirements (*B* = −0.534, *β* = −0.114, 95% CI: −0.883 to −0.185, *p* = 0.003), and expressive suppression (*B* = 0.801, *β* = 0.170, *p* < 0.001) were positively associated with total job burnout scores. The calculated coefficients for ICU/Emergency Department and Obstetrics/Pediatrics relative to Internal Medicine were (*B* = 3.643, *β* = 0.104, *p* = 0.024) and (*B* = 3.147, *β* = 0.084, *p* = 0.043), respectively. For contract-based employment, *p* = 0.057 in the multivariate model. The VI*F* values for each predictor variable in Model 3 ranged from 1.06 to 2.51, indicating no significant multicollinearity.

**Table 9:** Hierarchical multivariate linear regression analysis of the composite score for clinical nurse burnout.

Model	Variables	*B*	SE	95% CI	*β*	*t*	*p*	VIF
Model 3	Constant	65.553	7.143	[51.467, 79.640]		9.178	<0.001	
	Age	−0.080	0.079	[−0.237, 0.076]	−0.040	−1.010	0.314	1.30
	Monthly night shift	0.983	0.328	[0.336, 1.630]	0.163	2.996	0.003	2.51
	Gender (female = 1)	1.552	2.899	[−4.165, 7.268]	0.019	0.535	0.593	1.06
	Employment type (contract-based = 1)	2.002	1.045	[−0.060, 4.063]	0.069	1.915	0.057	1.10
	Department (surgical *vs*. medical)	−0.590	1.398	[−3.347, 2.168]	−0.018	−0.422	0.674	1.47
	Department (ICU/emergency *vs*. internal medicine)	3.643	1.597	[0.494, 6.791]	0.104	2.282	0.024	1.75
	Department (obstetrics/pediatrics *vs*. internal medicine)	3.147	1.544	[0.101, 6.192]	0.084	2.037	0.043	1.44
	Department (outpatient/Other *vs*. internal medicine)	2.837	1.678	[−0.473, 6.147]	0.079	1.691	0.093	1.86
	Cognitive reappraisal	−0.941	0.135	[−1.207, −0.675]	−0.299	−6.975	<0.001	1.56
	Expressive suppression	0.801	0.196	[0.414, 1.189]	0.170	4.082	<0.001	1.47
	Surface acting	0.958	0.132	[0.698, 1.218]	0.337	7.274	<0.001	1.82
	Deep acting	−1.018	0.233	[−1.479, −0.558]	−0.169	−4.363	<0.001	1.28
	Emotional expression requirements	−0.534	0.177	[−0.883, −0.185]	−0.114	−3.017	0.003	1.21

Continuous variables were included in the model as raw scores/raw units. *B* represents unstandardized coefficients, and *β* represents standardized coefficients. The Internal Medicine Department was used as the reference group. Model 1: *R*^2^ = 0.395, adjusted *R*^2^ = 0.371; Model 2: *R*^2^ = 0.656, adjusted *R*^2^ = 0.639, Δ*R*^2^ = 0.261, incremental *f*^2^ = 0.759; Model 3: *R*^2^ = 0.769, adjusted *R*^2^ = 0.754, Δ*R*^2^ = 0.113, incremental *f*^2^ = 0.487. Overall *p* < 0.001 for all models.

### 3.6 Analysis of the moderating effect of cognitive reappraisal

To examine whether cognitive reappraisal alters the strength of the association between surface acting and the composite score for job burnout, this study conducted a moderation analysis following control of covariates. Surface acting and cognitive reappraisal were first mean-centered, and then the “surface acting × cognitive reappraisal” interaction term was constructed. Expressive suppression, deep acting, emotional expression requirements, and complete department dummy variables were retained. The model including the interaction term had an R^2^ of 0.784, an adjusted *R*^2^ of 0.769, and an overall F value of 50.572 (*p* < 0.001).

The interaction term was statistically significant (*B* = −0.075, *β* = −0.128, 95% CI: −0.115 to −0.035, *p* < 0.001), contributing a Δ*R*^2^ of 0.015 and an incremental *f*^2^ of 0.070 compared to the model with the same covariates but without the interaction term. Simple slope analysis showed that when the cognitive reappraisal score was at the mean–1 SD, the mean, and the mean + 1 SD, the slopes for surface acting and the composite burnout score were *B* = 1.291 (95% CI: 0.983–1.599, *p* < 0.001), *B* = 0.964 (95% CI: 0.712–1.216, *p* < 0.001), and *B* = 0.637 (95% CI: 0.332–0.941, *p* < 0.001), respectively. As the level of cognitive reappraisal increased, the positive association between surface acting and job burnout gradually weakened.

The unique variance explained by the interaction term was approximately 1.5%, with an effect size of *f*^2^ = 0.070, indicating that the moderating role of cognitive reappraisal in the relationship between surface acting and occupational burnout is statistically significant, though its incremental contribution is relatively limited. Future studies could further evaluate the stability and practical implications of this moderating relationship through longitudinal tracking, multi-time-point measurements, or intervention studies. The detailed results regarding the moderating effect of cognitive reappraisal are shown in Table 10 and Figure 1.

**Table 10:** Analysis of the moderating effect of cognitive reappraisal on the association between surface acting and the composite score for job burnout.

Variables	*B*	SE	95% CI	*β*	*t*	*p*	VIF
Constant	68.624	5.963	[56.863, 80.384]		11.508	<0.001	
Age	−0.082	0.077	[−0.234, 0.069]	−0.041	−1.072	0.285	1.30
Monthly night shift	0.753	0.324	[0.114, 1.392]	0.125	2.323	0.021	2.60
Gender (female = 1)	1.388	2.809	[−4.153, 6.928]	0.017	0.494	0.622	1.06
Employment type (contract-based = 1)	1.905	1.013	[−0.094, 3.903]	0.066	1.880	0.062	1.11
Department (surgical *vs*. medical)	−0.936	1.358	[−3.615, 1.742]	−0.028	−0.689	0.491	1.48
Department (ICU/emergency *vs*. internal medicine)	3.763	1.548	[0.711, 6.816]	0.107	2.432	0.016	1.75
Department (obstetrics/pediatrics *vs*. internal medicine)	2.695	1.502	[−0.266, 5.657]	0.072	1.795	0.074	1.45
Department (outpatient/other *vs*. internal medicine)	2.246	1.634	[−0.977, 5.469]	0.063	1.374	0.171	1.88
Cognitive reappraisal (centralized)	−0.939	0.131	[−1.196, −0.681]	−0.299	−7.179	<0.001	1.56
Expressive suppression	0.780	0.190	[0.404, 1.155]	0.165	4.096	<0.001	1.47
Surface acting (centralization)	0.964	0.128	[0.712, 1.216]	0.339	7.552	<0.001	1.82
Deep acting	−1.089	0.227	[−1.536, −0.641]	−0.181	−4.795	<0.001	1.29
Emotional expression requirements	−0.557	0.172	[−0.895, −0.218]	−0.119	−3.244	0.001	1.21
Surface acting × cognitive reappraisal	−0.075	0.020	[−0.115, −0.035]	−0.128	−3.701	<0.001	1.08

Model *R*^2^ = 0.784, adjusted *R*^2^ = 0.769, interaction term Δ*R*^2^ = 0.015, F change = 13.698, *p* < 0.001, incremental *f*^2^ = 0.070; Maximum VIF = 2.60.

**Figure 1:**
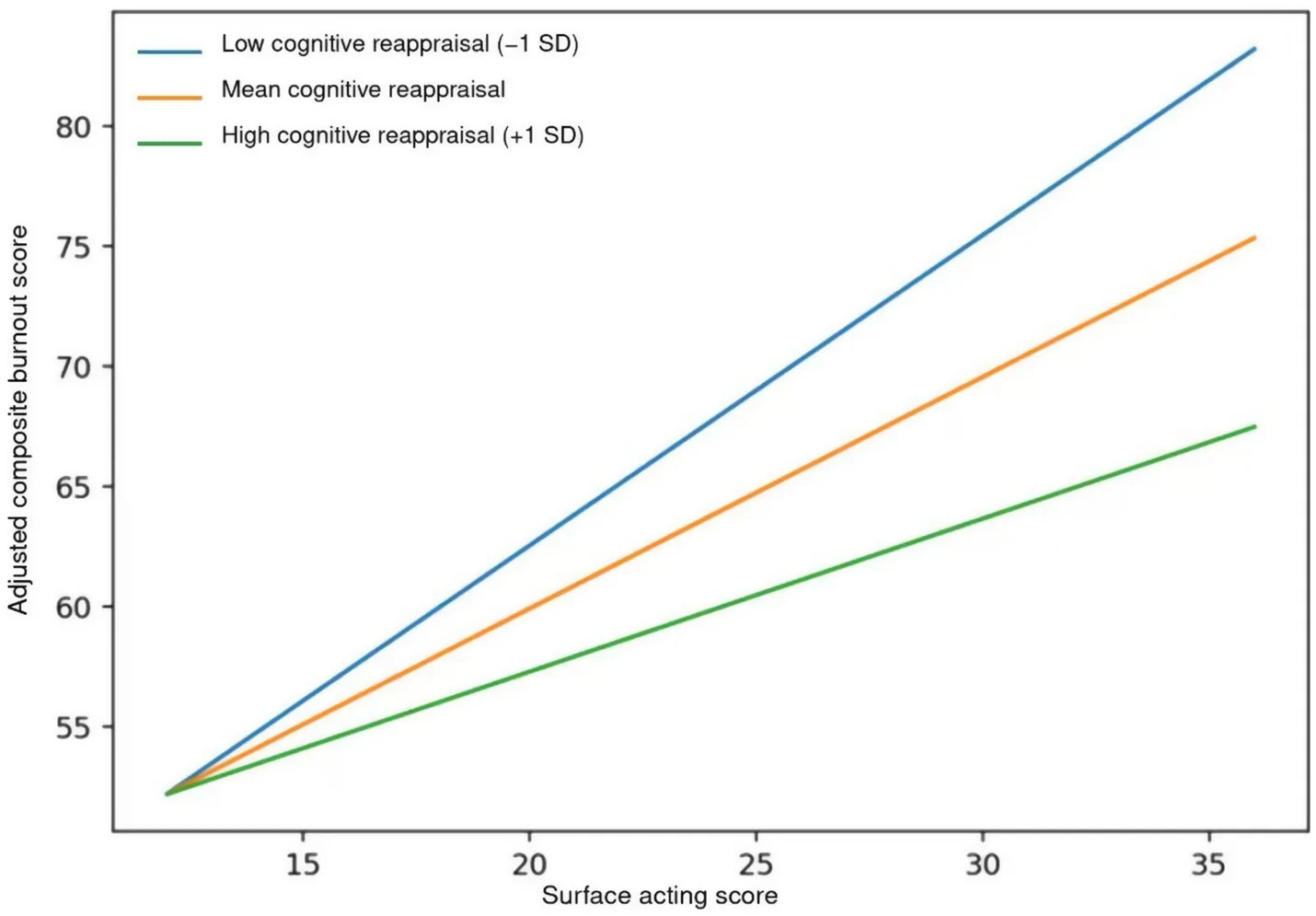
Simple slopes for surface acting and job burnout at different levels of cognitive reappraisal.

### 3.7 Regression diagnostics and sensitivity analysis

For Model 3, Durbin-Watson = 2.038. The Jarque-Bera test for residuals yielded *p* = 0.420, the Breusch-Pagan test yielded *p* = 0.477, and the Rainbow linearity test yielded *p* = 0.627. The corresponding statistics for the moderated model were Durbin-Watson = 2.100, Jarque-Bera *p* = 0.623, Breusch-Pagan *p* = 0.457, and Rainbow *p* = 0.423. Based on the residual distribution and VIF results, no obvious signs of violations of error independence, normality of residuals, homoscedasticity, linearity, or severe multicollinearity were found. It should be noted that the relatively high *R*^2^ was still partially influenced by common method variance from self-reports taken at the same time point. Therefore, mechanistic or causal interpretations should not be drawn based solely on the model’s explanatory power.

Sensitivity analyses using the three dimensions of the MBI-HSS as outcomes separately showed that the directions of association for the key psychological variables in Model 3 were generally consistent. When emotional exhaustion was used as the outcome, superficial acting had a B value of 0.521 (*p* < 0.001), cognitive reappraisal had a B value of −0.324 (*p* < 0.001), deep acting had a *B* = −0.284 (*p* = 0.012), and emotional expression requirements had a *B* = −0.186 (*p* = 0.031). When depersonalization was the outcome, the corresponding B values were 0.221 (*p* < 0.001), −0.157 (*p* < 0.001), −0.171 (*p* = 0.016), and −0.133 (*p* = 0.013), respectively. When low personal sense of accomplishment was the outcome, the corresponding B values were 0.216 (*p* < 0.001), −0.460 (*p* < 0.001), −0.564 (*p* < 0.001), and −0.215 (*p* = 0.004), respectively. These results support the directional stability of the research composite score analysis.

## 4 Discussion

This study observed among clinical nurses at a tertiary hospital in Jiangsu Province that different dimensions of emotional regulation strategies and emotional labor are associated with job burnout in varying directions and intensities. Cognitive reappraisal, deep acting, and emotional expression requirements were associated with lower composite scores for research-based occupational burnout, while expressive suppression and surface acting were associated with higher scores. Among these, surface acting showed a relatively strong correlation with job burnout. The above results indicate that job burnout among nurses is not only related to workload and job characteristics but is also closely linked to their emotional regulation strategies and the emotional management requirements of their organizations.

Cognitive reappraisal showed a strong negative correlation with the composite score for job burnout and maintained this negative association in the multivariate model. Previous theoretical work suggests that cognitive reappraisal contributes to emotional regulation by altering the way situations are interpreted (4–6), providing a theoretical framework for the findings of this study. However, this study could not determine whether higher levels of cognitive reappraisal precede lower levels of burnout or whether individuals with lower burnout are more likely to use cognitive reappraisal. Nor could it rule out the combined influence of unmeasured factors such as personality and organizational support. Therefore, the practical significance of cognitive reappraisal should be understood as a research lead worthy of further validation, rather than a proven intervention target.

Expressive suppression was positively correlated with the composite score for job burnout and maintained an independent association even in the fully adjusted model. Relevant literature indicates that sustained suppression of experienced emotions is associated with higher cognitive and physiological loads (9, 10). However, this study did not measure the specific contexts in which suppression occurred, its duration, or organizational emotional regulations. Nor could it determine whether expressive suppression preceded burnout chronologically. Therefore, this study treats it solely as an emotion-regulation characteristic that co-occurs with higher levels of burnout and refrains from making causal inferences.

The correlation between surface acting and the composite score for job burnout was the strongest in this study (r = 0.714) and also showed a positive association in the full regression model. Surface acting emphasizes the inconsistency between external display and internal feelings. The theoretical link between this construct and emotional exhaustion has been discussed in previous studies (11–13, 17). However, the high correlation coefficient must be interpreted with caution due to common method bias, as both surface acting and burnout were self-reported by the same participants at the same time point, and the identical measurement method amplifies covariance. Therefore, the high *R*^2^ and strong correlation cannot stand alone as causal evidence.

In this study, emotional expression requirements were negatively correlated with the composite score for job burnout and remained negatively associated even after controlling for demographic characteristics, emotional regulation strategies, and other dimensions of emotional labor. Emotional expression requirements reflect the extent to which a hospital or position regulates nurses’ emotional displays, service attitudes, and communication behaviors. The relationship between these requirements and job burnout is not simply a linear function of the intensity of the requirements but is instead jointly influenced by work context factors such as the clarity of rules, the appropriateness of the requirements, and the adequacy of organizational support.

When emotional expression requirements fall within a reasonable range and are supported by appropriate organizational resources, their association with lower levels of job burnout can be understood from the following perspectives. First, clear norms for emotional behavior provide nurses with a behavioral reference during conflicts, high-stress situations, or complex nurse–patient interactions, thereby reducing the additional psychological strain caused by role ambiguity and situational uncertainty. Second, if organizational requirements emphasize professional communication, empathy, and internal cognitive adjustment, rather than merely focusing on external expression management, it would be more conducive to promoting deep acting, aligning internal experiences with professional expressions, and reducing long-term emotional dysregulation. Third, emotional expression norms consistent with nursing ethics help clarify professional role boundaries, making emotional management an integral part of clinical communication skills and professional competence, thereby strengthening professional identity and a sense of work meaning. Furthermore, relatively consistent and moderate expression rules help establish stable expectations for interactions between nurses and patients as well as among colleagues, thereby reducing unnecessary interpersonal conflicts. When emotional expression norms are complemented by communication training, psychological support, team support, and managerial support, nurses are better equipped to develop effective emotional coping strategies and stress regulation abilities. These mechanisms align with the dynamic balance between work demands and individual and organizational resources emphasized by the JD-R theory and Grandey’s emotional regulation framework (3, 7–9).

Analysis of demographic and job characteristics revealed differences in univariate comparisons for age, department, professional title, employment status, and night shift grouping. Tukey’s HSD test further identified specific differences between certain groups. The full regression analysis used k-1 dummy variables for departments. The results showed that ICU/Emergency and Obstetrics/Pediatrics still had higher composite scores than Internal Medicine in the final regression model, while the adjusted differences for Surgery and Outpatient/Other were not statistically significant. Contract-based employment scored higher in univariate comparisons, but the *p*-value was 0.057 after multivariate adjustment. With only 6 male participants, there was insufficient statistical power for comparisons between gender groups. Therefore, this study does not conclude that “there is no difference by gender.” Moderation analysis revealed that as the level of cognitive reappraisal increased, the positive slope between surface acting and the composite burnout score gradually decreased. The interaction term added approximately 1.5% to the explanatory power in the full covariate model (f^2^ = 0.070). Although the incremental contribution was smaller than the main effects of surface acting and cognitive reappraisal, this result indicates that individual emotional regulation styles influence the strength of the association between surface acting and occupational burnout. Future studies could employ longitudinal tracking, repeated-measure, or intervention designs in multicenter samples to further examine the stability and practical implications of this moderating relationship.

This study still has certain limitations. First, the cross-sectional design primarily reflects the associations among variables at a single point in time. The temporal sequence and mechanisms of action require further confirmation through longitudinal studies. Second, the participants were recruited from a single-center tertiary hospital using convenience sampling, and the number of male nurses was small. Therefore, caution should be exercised when generalizing the findings to other regions and different types of medical institutions. Third, all key variables were measured using self-report questionnaires. Although Harman’s single-factor test did not reveal any significant single-factor dominance, common method variance still potentially influenced the associations among variables. Fourth, sample size was determined primarily based on empirical rules for multiple regression. The statistical power of the moderation analysis requires further validation in a larger sample. Fifth, the three subscales of the MBI-HSS have relatively independent theoretical meanings. The composite burnout score constructed in this study is primarily used for continuous variable analysis and should still be interpreted in conjunction with the results of each subscale. Sixth, this study did not further measure the clarity and appropriateness of emotional expression requirements or the corresponding level of organizational support. Future research may include variables such as organizational emotional rules, psychological safety, and managerial support to further analyze their relationship with job burnout.

## 5 Conclusion

There is a close association between clinical nurses’ emotional regulation strategies, emotional labor styles, and levels of job burnout. Cognitive reappraisal, deep acting, and emotional expression requirements are associated with lower composite job burnout scores, while expressive suppression and surface acting are associated with higher composite job burnout scores. Among these, surface acting shows a particularly strong correlation with job burnout. Work characteristics such as the frequency of night shifts and department assignment are also associated with levels of job burnout.

Further analysis revealed that cognitive reappraisal plays a significant moderating role in the relationship between surface acting and job burnout. When cognitive reappraisal levels are high, the positive association between surface acting and job burnout is relatively weakened. The association between emotional expression requirements and lower occupational burnout scores suggests that clear, moderate, and nursing-profession-compliant emotional expression requirements may have positive implications for organizational management. In nursing management, priority should be given to groups of nurses who frequently engage in surface acting, experience significant emotional suppression, and bear a heavy night-shift workload. At the same time, emphasis should be placed on the coordinated development of cognitive reappraisal, deep emotional regulation, professional emotional norms, and organizational support to promote nurses’ occupational mental health and the stability of the nursing workforce.

## Data Availability

The raw data supporting the conclusions of this article will be made available by the authors, without undue reservation.
